# Free-Standing PVDF/Reduced Graphene Oxide Film for All-Solid-State Flexible Supercapacitors towards Self-Powered Systems

**DOI:** 10.3390/mi11020198

**Published:** 2020-02-14

**Authors:** Parthiban Pazhamalai, Vimal Kumar Mariappan, Surjit Sahoo, Woo Young Kim, Young Sun Mok, Sang-Jae Kim

**Affiliations:** 1 Nanomaterials and System Lab, Major in Mechatronics Engineering, Faculty of Applied Energy System, Jeju National University, Jeju 63243, Korea; parthiban.selvam09@gmail.com (P.P.); vimalece10@gmail.com (V.K.M.); surjit488@gmail.com (S.S.); 2 Department of Electronic Engineering, Faculty of Applied Energy System, Jeju National University, Jeju 63243, Korea; semigumi@jejunu.ac.kr; 3 Department of Chemical and Biological Engineering, Jeju National University, Jeju 63243, Korea; smokie@jejunu.ac.kr; 4 Department of Advanced Convergence Science and Technology, Jeju National University, Jeju 63243, Korea

**Keywords:** polyvinylidene fluoride, polymer nanocomposites, flexible supercapacitors, electrospinning, self-powered system

## Abstract

The development of polymer-based devices has attracted much attention due to their miniaturization, flexibility, lightweight and sustainable power sources with high efficiency in the field of wearable/portable electronics, and energy system. In this work, we proposed a polyvinylidene fluoride (PVDF)-based composite matrix for both energy harvesting and energy storage applications. The physicochemical characterizations, such as X-ray diffraction, laser Raman, and field-emission scanning electron microscopy (FE-SEM) analyses, were performed for the electrospun PVDF/sodium niobate and PVDF/reduced graphene oxide composite film. The electrospun PVDF/sodium niobate nanofibrous mat has been utilized for the energy harvester which shows an open circuit voltage of 40 V (peak to peak) at an applied compressive force of 40 N. The PVDF/reduced graphene oxide composite film acts as the electrode for the symmetric supercapacitor (SSC) device fabrication and investigated for their supercapacitive properties. Finally, the self-charging system has been assembled using PVDF/sodium niobate (energy harvester), and PVDF/reduced graphene oxide SSC (energy storage) and the self-charging capability is investigated. The proposed self-charging system can create a pathway for the all-polymer based composite high-performance self-charging system.

## 1. Introduction

The extensive use of renewable energy/power sources paves the way for the growing energy crisis and the environmental pollution issues, which creates the demand towards the need for energy sources [1,2]. To overcome the energy crisis, many researchers are focused on developing new energy devices based on inorganic materials and their composites [3,4,5,6]. From the available literature, the inorganic composite materials have a higher advantage due to their synergetic effect arising from the composite materials, which show enhanced performances [7]. The composition, including the carbon and carbon derivatives composites with the metal oxides and sulfides, have received considerable attention in the field of energy [8]. However, the rigid nature of the composite materials makes them difficult to apply in flexible and lightweight electronics applications. For developing flexible and lightweight materials, polymer nanocomposites attracted much compared to the other conventional composites due to their improved property and performance [9]. In recent reports, the polymer composites have high flexibility, hydrophilicity/hydrophobicity, thermal stability, gas barrier performance, optical clarity, decreased flammability, and enhanced mechanical properties making them the top candidate for various applications [10]. These polymer-inorganic composite materials consist of nanosized inorganic particles which are dispersed uniformly in a polymer matrix. The major advantages of these polymer composite films are ease of synthesis, lightweight, flexible, and corrosion resistance [11]. The main benefits of the composite film over the pure polymer is the enhancement in the electrical, mechanical and tensile properties of the polymer composites [12,13]. The enhancement in the properties of the composite film is mainly due to the interaction between the filler materials and the carbon chain groups in the polymer matrix [14,15]. Over several classes of polymers available, polyvinylidene fluoride (PVDF) is a highly non-reactive thermoplastic fluoropolymer produced by the polymerization of vinylidene difluoride which has high resistance towards the variety of solvents(acids, and bases) and has a low density compared to other fluoropolymers [16]. PVDF is commonly used in the chemical, semiconductor, medical, and defense industries, as well as in electrochemical energy storage (separator and binders) and energy-harvesting applications [17]. PVDF membranes are also used for Western blots for the immobilization of proteins due to its non-specific affinity for amino acids [18]. The researchers have also discovered the energy-harvesting piezoelectric properties of PVDF and exploited their use as piezoelectric nanogenerators to power tactile sensors, inexpensive strain gauges, and lightweight audio transducers [19,20]. PVDF is also used in the field of energy storage as the standard binder for the preparation of composite electrodes for electrochemical energy storage and as the separator since it is chemically inert over a wide potential range and does not react with the electrolyte in the system [21]. Due to the nature of the PVDF and its extensive properties in the field of energy, PVDF incorporated with the nanomaterials can be applied to the field of wearable and flexible energy harvesting and storing applications. The utilization of the sodium niobate cubes in the PVDF for the piezoelectric nanogenerator is as follows: (i) sodium niobate is a lead-free piezoceramic with biocompatible in nature; (ii) bulk production of sodium niobate nanocubes; (iii) ease of large-scale fabrication of device with low cost; (iv) the electric field can effectively pole random piezoelectric domains to one direction due to the ferroelectric nature of the sodium niobate; (v) PVDF polymer plays a major role of preventing the cracking and breaking of sodium niobate cubes upon mechanical strain which helps in long cycle life, and (vi) its flexible nature which increases the volume fraction of the sodium niobate in the polymer matrix results in high piezoelectric response. For the above reasons, we utilized sodium niobate cubes in the PVDF matrix for the piezoelectric nanogenerator application. In this work, we incorporated sodium niobate cubes in the PVDF polymer matrix and fabricated the piezoelectric nanofibers via electrospinning for the lightweight piezoelectric energy-harvesting application, and for the energy storage, graphene in the PVDF matrix to form the flexible conductive film as the electrode for a supercapacitor application. The fabricated electrospun PVDF/sodium niobate nanogenerator and the PVDF/reduced graphene oxide symmetric supercapacitor (SSC) was coupled to form a self-charging system in which the flexible power generator can charge the supercapacitor under the applied compressive mechanical force. The above mentioned findings can provide new insight into the development of flexible self-charging electronics applications.

## 2. Materials and Methods

### 2.1. Materials

Graphite powder and polyvinylidene fluoride (PVDF) were procured from Sigma Aldrich Ltd. Niobium oxide (Nb_2_O_5_), potassium permanganate (KMnO_4_), sulfuric acid (H_2_SO_4_), hydrogen peroxide (H_2_O_2_), hydrochloric acid (HCl), sodium hydroxide (NaOH), hydrazine hydrate (assay 50%), polyvinyl alcohol (PVA), phosphoric acid (H_3_PO_4_), N,N-dimethylacetamide and acetone were obtained from Dae Jung Chemicals and Metal Co., Ltd. (Gyeonggi-do, Korea). All the chemicals used in this research were research-grade, and double distilled water was used throughout the experiments.

### 2.2. Preparation of Sodium Niobate Nanocubes

A facile hydrothermal technique was employed for the preparation of sodium niobate nanocubes. Briefly, an aqueous solution of 240 mM sodium hydroxide was prepared in 60 mL of double distilled water [22]. We added 3.76 mM of niobium oxide to the sodium hydroxide solution and stirred for 1 h until complete dissolution to form a homogeneous solution. The prepared solution was then transferred to a 100 mL Teflon lined stainless steel autoclave and kept at 150 °C for 10 h. After the completion of the hydrothermal reaction, the obtained product was centrifuged with doubly distilled water several times and dried at 80 °C in a hot air oven. The final product was annealed further at 600 °C for 12 h to obtain pure sodium niobate nanocubes. The reaction mechanism in the formation of sodium niobate is due to orientation attached with an Ostwald ripening process as reported in the earlier works [23,24].

### 2.3. Electrospinning of Polyvinylidene Fluoride (PVDF)/Sodium Niobate Nanofibers

A facile and low-cost electrospinning technique was adopted for the preparation of composite PVDF/sodium niobate nanofibers. Briefly, the PVDF polymer solution was prepared as per the literature [13]. At first, 10 wt % of PVDF was dissolved in acetone and N,N-dimethylacetamide in the ratio of 70:30 (V/V), then further ultra-sonicated to form the transparent polymer solution. To the prepared PVDF solution, 10 wt% of the sodium niobate was added and stirred vigorously for 48 h to form a white-colored viscous solution. The obtained viscous solution is loaded on to a 15 mL syringe and placed in the electrospinning setup with a stainless-steel needle size of 25 G at a flow rate of 0.5 mL/h under a constant voltage of 15 KV with the tip to collector distance 10 cm to obtain PVDF/sodium niobate nanofibrous mat. The obtained electrospun mat was dried in a vacuum oven at 60 °C for 24 h for stabilization and to remove surface water. After the stabilization process, the mat as used for the fabrication of a nanogenerator device. The nanogenerator was fabricated using an electrospun mat of dimension 2 × 2 cm^2^ with Al as the top and bottom electrodes. Cu wires were attached with silver paste to the top and bottom electrodes to establish the electrical connections. The fabricated device was used for further characterization.

### 2.4. Preparation of Graphene Nanosheets

A simple modified Hummers method followed by the sonochemical technique was adopted for the preparation of graphene nanosheets [25]. Initially, graphene oxide (GO) sheets were prepared using graphite powder, H_2_SO_4_, KMnO_4_, H_2_O_2_, and HCl as the starting precursor according to the modified Hummers method as stated in our earlier reports. A simple sonochemical reaction was adopted for the preparation of graphene nanosheets using prepared GO as the starting material [25]. Concisely, 0.2 g of GO is suspended in 200 mL of the double-distilled water, and the pH of the GO solution was adjusted by the addition of NaOH solution to reach the pH value of 10 followed by the addition of hydrazine hydrate (2 mL) which acts as the reducing agent. The prepared solution subjected to ultrasound irradiation for 5 h under ambient conditions. After completion of the reaction, the resulted graphene nanosheets were centrifuged several times using distilled water until the product is free from a trace amount of residual impurities and dried in a hot air oven at 80 °C before further characterization.

### 2.5. Preparation and Fabrication of PVDF/Reduced Graphene Oxide Solid-State Supercapacitor

The PVDF/reduced graphene oxide composite film electrode was prepared using a simple solution casting technique. A 25 wt % of prepared graphene nanosheet was added to the prepared PVDF solution and ultra-sonicated until the graphene nanosheets are finely dispersed in the polymeric solution. The graphene nanosheet dispersed polymer solution was poured onto the pre-cleaned petri dish and kept in a hot air oven at 60 °C for 24 h. After complete drying, the composite film was used as the electrode for the supercapacitor. The electrical conductivity of the prepared PVDF/reduced graphene oxide film is around 2 S/cm. The polymer gel electrolyte composed of PVA and H_3_PO_4_ was prepared using the method previously reported in the literature [26]. Briefly, 5 g of PVA is dissolved in 50 mL of water using mechanical stirring at a temperature of 80 °C. After the complete dissolution of PVA, a transparent solution was obtained to which 5 g of H_3_PO_4_ was added and allowed to vigorous stirring under heat until a transparent gel was formed. Finally, the prepared PVA/H_3_PO_4_ polymer gel was used for the fabrication of a supercapacitor device.

The supercapacitor device was fabricated using the prepared PVDF/reduced graphene oxide composite film as the electrode for the device with PVA/H_3_PO_4_ as the electrolyte. The gel electrolyte was coated to the electrode surface of dimension 1.0 × 1.0 cm^2^, and then two electrodes were sandwiched to form as the supercapacitor device. The electrochemical analysis of the fabricated supercapacitor device was examined via cyclic voltammetry (CV) and galvanostatic charge-discharge (CD) measurements using an Autolab PGSTAT302N electrochemical workstation.

### 2.6. Instrumentation

The phase formation and the crystallinity of the graphene nanosheets and sodium niobate nanostructures were determined using the Rigaku X-ray diffractometer operated at 40 keV and 40 mA Cu Kα radiation. The ultrasound irradiation used for the dissolution of PVDF polymer solution was carried out on a SONIC (Newtown, CT, USA) VCX 500 system (20 kHz, 500 W) with the aid of direct immersion titanium (Ti-6Al-4V) horn. The Raman spectra of the PVDF/reduced graphene oxide composite film and sodium niobate were obtained using Lab Ram HR Evolution Raman spectrometer (Horiba Jobin-Yvon, France), operated at 10 mW laser power with an excitation wavelength of 514 nm with an Ar^+^ ion laser. The data were collected using a 10 s data point acquisition time. The PVDF/sodium niobate nanofibers were prepared to utilize the electrospinning/spray system (NanoNC; Model: ESR200R2, Seoul, Korea). The surface morphology of the PVDF/reduced graphene oxide composite film and electrospun PVDF/sodium niobate mat were analyzed using a field-emission scanning electron microscope (FE-SEM, JSM-6700F, JEOL Ltd., Tokyo, Japan). The external force applied to the nanogenerator device using a linear motor (E1100, LinMot, USA) and the open-circuit voltage and the short-circuit current were recorded using nano voltmeter (Keithley 2182A, Cleveland, OH, USA) and a picoammeter (Keithley 6485), respectively.

## 3. Results and Discussion

**Figure 1:**
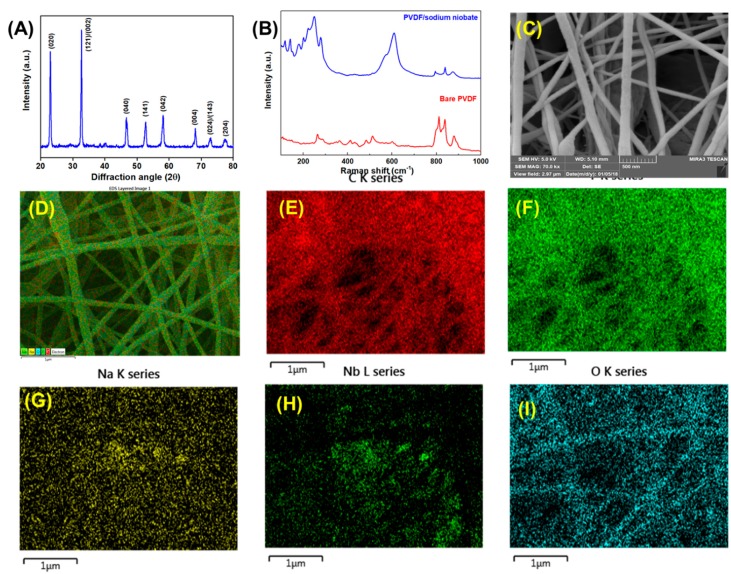
(**A**) X-ray diffraction pattern of the prepared sodium niobate. (**B**) Laser Raman spectra of the bare polyvinylidene fluoride (PVDF) and electrospun PVDF/sodium niobate nanofibrous mat. Surface morphology of the PVDF/sodium niobate nanofibers (**C**) high-resolution micrographs. Elemental mapping analysis of the PVDF/sodium niobate nanofibers (**D**) overlay field-emission scanning electron micrograph (FE-SEM) of PVDF/sodium niobate nanofibers, (**E**) elemental mapping showing the distribution of C element, (**F**) elemental mapping showing the distribution of F element, (**G**) elemental mapping showing the distribution of Na element, (**H**) elemental mapping showing the distribution of Nb element and (**I**) elemental mapping showing the distribution of O element present in PVDF/sodium niobate nanofibers.

The physicochemical characterization of the sodium niobate and PVDF/sodium niobate nanofibers are provided in Figure 1. Figure 1A shows the distinct diffraction pattern of the crystalline phase of sodium niobate well matched with the JCPDS no.: 82-0606 [22]. The peak obtained at the diffraction angle 22.94°, 32.75°, 46.8°, 52.67°, 58.11°, 68.2°, 72.85°, and 77.41° corresponds to the (020), (121)/(002), (040), (141), (042), (004), (024)/(143), and (204) planes of the sodium niobate with the space group of *P21ma*. Figure 1B and Figure S1 shows the Raman spectrum of the electrospun PVDF, PVDF/sodium niobate nanofibrous mat, and as prepared sodium niobate. The electrospun PVDF mat indicates the presence of significant bands over 790 to 900 cm^−1^ [27] whereas the PVDF/sodium niobate shows the major vibrational bands at 115, 140, 175, 198, 224, 246, 276, 428, 571, 612, and 870 cm^−1^ all correspond to the ferroelectric phase of sodium niobite [28]. The presence of a Raman band observed at 615 cm^−1^ (ν_1_) accompanied by a shoulder at around 571 cm^−1^ (ν_2_) confirms the orthorhombic perovskite structure of the sodium niobate in the PVDF matrix [22]. Furthermore, the vibration bands due to PVDF are diminished in the composite since the vibration bands that have arisen from crystalline sodium niobate overwhelm the weak vibrations bands of PVDF [29]. The surface morphology and the elemental mapping analysis of the bare sodium niobate and the electrospun PVDF/sodium niobate are shown in Figure S2 and Figure 1B. Figure 1C shows the FE-SEM micrograph of electrospun PVDF/sodium niobate which clearly depicts the formation of nanofibers with an average diameter ranging from 100–200 nm. Furthermore, the elemental mapping of the electrospun PVDF/sodium niobate nanofibrous mat (Figure 1D–I) shows the uniform distribution of Na, Nb, O, C, and F in the prepared nanofibrous mat. 

**Figure 2:**
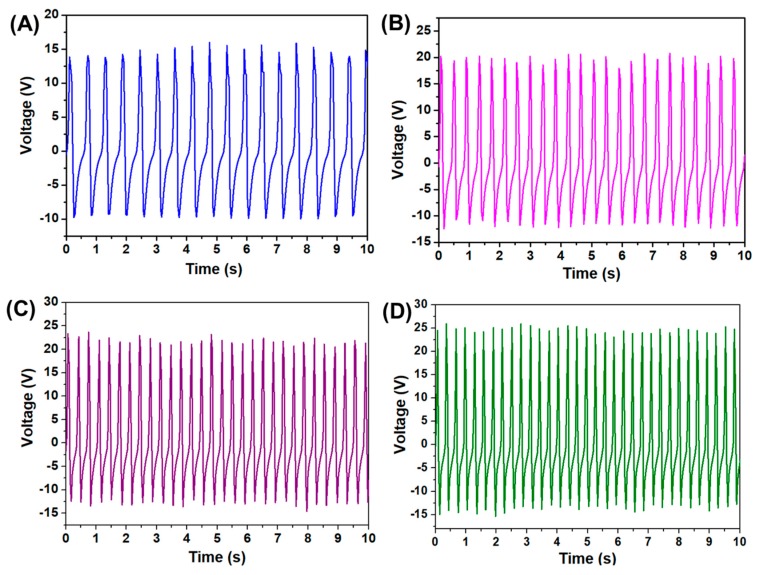
(**A**–**D**) the voltage output of PVDF/sodium niobate nanogenerator device measured under various compressive forces (10 to 40 N).

The piezoelectric characterization of the bare PVDF and electrospun PVDF/sodium niobate nanofibrous mat was investigated by fabricating the nanogenerator device [30]. Figure S3 shows the comparative voltage output of the PVDF and electrospun PVDF/sodium niobate nanofibrous mat obtained under an applied compressive force of 10 N. The incorporation of sodium niobate in the PVDF matrix results in an output voltage of 25 V, which is higher compared to bare PVDF, thus indicating the enhanced piezoelectric response. The improved energy harvesting properties of the PVDF/sodium niobate is due to the enhancement in dipole alignments of PVDF and the synergetic effect of the piezoelectric ceramics [13,31]. The voltage output of the fabricated device under various compressive forces (10 to 40 N) is shown in Figure 2A–D. The output peak to peak voltage of the electrospun PVDF/sodium niobate nanofibrous mat is about 25 V under an applied compressive force of 10 N. With the increase in the applied compressive force, the peak to peak voltage of about 40 V is obtained for the fabricated energy harvester. The increased voltage output from 25 to 40 V with an increase in the applied compressive force of 40 N is due to the enhancement in the piezo-response of the PVDF/sodium niobate mat [30,32]. The comparative voltage output of the various piezoelectric nanogenerator is compared with the current work and tabulated in Table 1. 

From the obtained results, it is evident that the output of the PVDF/sodium niobate nanogenerator is highly dependent on the applied compressive force and directly proportional to its piezo-voltage. The electrospun PVDF/sodium niobate mat as the active piezoelectric layer shows enhanced output compared with the other reported piezoelectric nanogenerators and the improved performance is due to the following reasons: (i) high ferroelectric property of the PVDF and sodium niobate results in the enhancement of the piezoelectric property of the composite, (ii) high voltage applied in the fabrication methodology, which enhances the arrangement of dipoles in the prepared composite mat [38,39]. These results highlight that the electrospun PVDF/sodium niobate nanofibrous nanogenerator with improved piezoelectric output will be a promising candidate as a power source for the self-charging systems.

**Figure 3:**
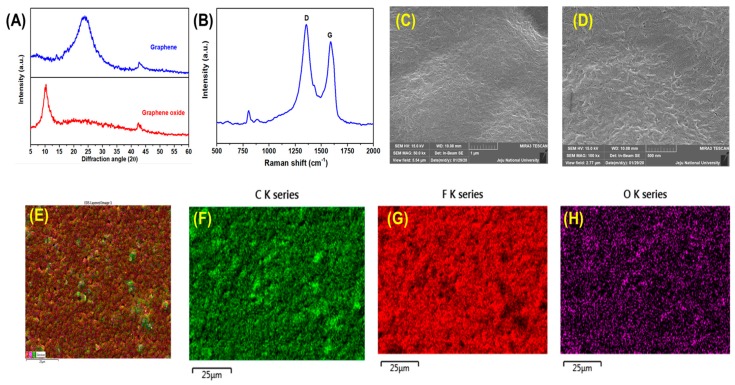
(**A**) X-ray diffraction pattern of the graphene and graphene oxide. (**B**) Laser Raman spectrum of the PVDF/reduced graphene oxide composite film. Surface morphology of the PVDF/reduced graphene oxide composite film, (**C**) low resolution, and (**D**) high-resolution micrographs. Elemental mapping analysis of the PVDF/reduced graphene oxide composite film (**E**) overlay field-emission scanning electron micrograph of PVDF/reduced graphene oxide composite film, (**F**) elemental mapping showing the distribution of C element, (**G**) elemental mapping showing the distribution of F element, (**H**) elemental mapping showing the distribution of O element present in PVDF/reduced graphene oxide composite film.

In the view of energy storage application, PVDF/reduced graphene oxide has been chosen as the electrode material. Figure 3 shows the physico-chemical characterization of the prepared graphene and PVDF/reduced graphene oxide composite film. Figure 3A shows the X-ray diffraction pattern for the prepared graphene oxide and reduced graphene oxide. The diffraction pattern of the graphene oxide shows a sharp predominant peak at 10.4° correspond to the (001) plane of the carbon present in the graphene oxide whereas, after the sonochemical process, the graphene oxide was reduced entirely to graphene nanosheets with a sharp and broad peak at 24.3° assigned to the (002) plane which suggest the increase in the graphitic nature of the material [40]. Figure 3B shows the Raman bands of the PVDF/reduced graphene oxide composite film exhibits the characteristic Raman vibrational bands of graphene. The band at 1357 and 1599 cm^−1^ corresponds to the D and G band of the graphene in the PVDF matrix [41], and the other bands in the region of 700 to 1000 cm^−1^ corresponds to the characteristic band of PVDF [42]. The surface morphology and the elemental mapping analysis of PVDF/reduced graphene oxide composite film are shown in Figure 3C–G. Figure S4A,B show the FE-SEM micrograph of reduced graphene oxide resembling the sheet like morphology. Figure S4C–F shows the elemental mapping analysis of the reduced graphene oxide confirms the presence of carbon and oxygen uniformly distributed in the sheets. The energy-dispersive X-ray spectroscopy (EDS) spectrum reveals the reduced graphene oxide contains C:O in the ratio of 84.2:15.8 which is in agreement with the previous reports [43]. The FESEM micrograph shown in Figure 3C,D shows the low and high-resolution micrograph of the PVDF/reduced graphene oxide composite film with the uniform distribution of the reduced graphene oxide in the PVDF composite [44]. Besides, the elemental mapping analysis provided in Figure 3E–H also confirms the presence of carbon, oxygen, fluorine in the PVDF/reduced graphene oxide composite film. 

**Figure 4:**
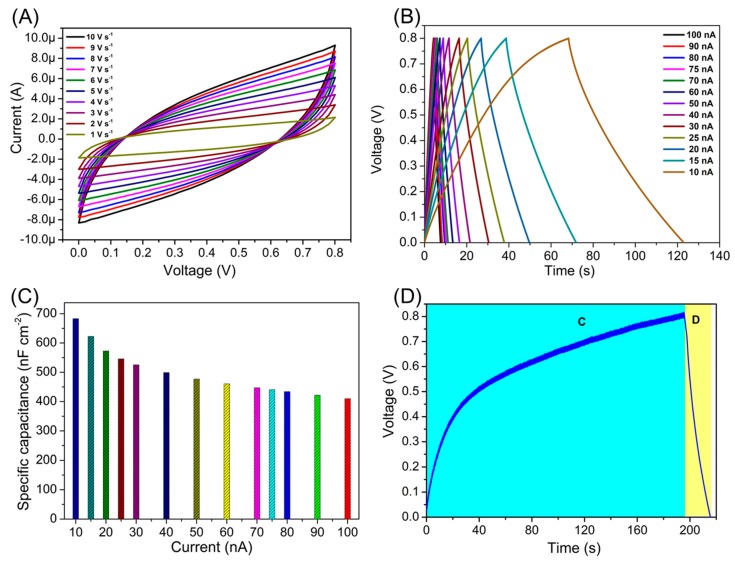
Electrochemical characterization of the PVDF/reduced graphene oxide symmetric supercapacitor (SSC). (**A**) Cyclic voltammetry profiles of PVDF/reduced graphene oxide SSC recorded using various scan rates (1–10 V s^−1^). (**B**) Galvanostatic charge-discharge profiles of the PVDF/reduced graphene oxide SSC recorded using different current (10 to 100 nA). (**C**) Effect of current on the specific capacitance of the PVDF/reduced graphene oxide SSC. (**D**) The charging profile of the PVDF/reduced graphene oxide SSC with the applied compressive force of 40 N applied to the PVDF/sodium niobate nanogenerator device.

The electrochemical properties of the prepared PVDF/reduced graphene oxide film were investigated by fabricating a symmetric solid-state supercapacitor device using PVDF/reduced graphene oxide as the electrode and PVA/H_3_PO_4_ as the electrolyte. Initially, the cyclic voltammetric analysis for the fabricated PVDF/reduced graphene oxide SSC was recorded over the voltage window of 0–0.8 V measured at various scan rates ranging from 1 to 10 V s^−1^ as shown in Figure 4A. The CV profiles show the characteristics of rectangular-shaped curves suggesting the charge storage in the PVDF/reduced graphene oxide SSC is due to the formation of double-layer capacitance [45]. The quasi-rectangular shape of the CV profiles retains its shape over the scan rate (1 to 10 V s^−1^) symbolizing the excellent capacitive nature of the PVDF/reduced graphene oxide SSC device. The current of the CV profiles is increasing with the increase in the scan rate, suggesting the better capacitive nature of the device [46]. The galvanostatic CD profiles of the PVDF/reduced graphene oxide SSC device were measured over the voltage window of 0–0.8 V at various current ranging from 10 to 100 nA, as shown in Figure 4B. The PVDF/reduced graphene oxide SSC device displays quasi-triangular-shaped CD profiles, which are in agreement with the CV profiles shown in Figure 4A suggesting the mechanism of charge storage is due to the electrostatic non-faradaic process as observed in the carbon-based supercapacitors [47]. The CD profile retained its symmetric behavior at all the current densities, suggesting better capacitive property of the PVDF/reduced graphene oxide SSC device [48]. From Figure 4B it is evident that the device undergoes fast charging and discharging at higher current, whereas at the lower current, better charging and discharging profiles were obtained for the PVDF/reduced graphene oxide SSC device [49]. The specific capacitance (*C*) of the PVDF/reduced graphene oxide SSC device was calculated using the relation [39]:

*C = I × Δt/ΔV × A* (1)

Here “*I*” is the discharge current (A), “*Δt*” is the discharge time (s), “*ΔV*” is the voltage window (V), and “*A*” is the area of the PVDF/reduced graphene oxide SSC device the electrode (cm^−2^). The PVDF/reduced graphene oxide SSC device delivered a specific capacitance of 683 nF was calculated from the CD profile measured at a constant discharge current of 10 nA. The change in the specific capacitance of the PVDF/reduced graphene oxide SSC device with respect to the current is provided in Figure 4C. A specific capacitance of 410 nF cm^−2^ was obtained at a higher current density of 50 nA cm^−2,^ which almost retained 69.69 % of its initial capacitance with an increase of 5-fold in the current suggesting the better rate capability of the PVDF/reduced graphene oxide SSC device [50,51]. Figure S5 shows the Coulombic efficiency plot of the PVDF/reduced graphene oxide SSC as a function of current. The PVDF/reduced graphene oxide SSC shows coulombic efficiency over 80% for all the current ranges suggesting the better charging/discharging rate compared to the other reported supercapacitors [50,52]. Figure S6 shows the cyclic performance of the PVDF/reduced graphene oxide SSC device examined using galvanostatic CD analysis using a constant current of 50 nA over 10,000 cycles. The PVDF/reduced graphene oxide SSC device exhibits capacitance retention of about 87% to initial capacitance of charge-discharge, suggesting the better stability of the PVDF/reduced graphene oxide SSC device. 

To investigate the capability of the self-charging system as a practical power source for electronic devices, the PVDF/sodium niobate nanogenerator was integrated with the PVDF/reduced graphene oxide SSC device to form the self-charging power system as shown in the scheme provided in Figure S7. The output from the PVDF/sodium niobate nanogenerator is a pulsed AC signal, and hence a bridge rectifier was used to convert the generated AC output of the nanogenerator from alternating current (AC) to direct current (DC) before charging the PVDF/reduced graphene oxide SSC device [53]. The charging curve of the PVDF/reduced graphene oxide SSC under a compressive force of 40 N and discharged using a constant current of 25 nA was provided in Figure 4D. The PVDF/reduced graphene oxide SSC took 190 s to charge to 0.8 V under a constant compressive force of 40 N and discharged using a constant current. The time taken to charge the PVDF/reduced graphene oxide SSC is better compared to the previously reported self-powered supercapacitors, as listed in Table 2. 

The charging behavior of the PVDF/reduced graphene oxide SSC connected with the PVDF/sodium niobate nanogenerator under various applied compressive force is provided in Figure S8. The comparison of the time to charge versus the applied compressive force is provided in Figure S9. It provides clear evidence that the charging of the PVDF/reduced graphene oxide SSC mainly depends upon applied compressive force as the higher force the charging time is lesser whereas, the lower the force, the higher the time to charge. The charging time increases with the decrease in the applied force to the nanogenerator device is the phenomenon that the piezo-output is directly proportional to an applied force. The stored charge can be utilized for the small electronic application in day-to-day life.

## 4. Conclusions

In this work, we successfully fabricated the PVDF/reduced graphene oxide electrode and electrospun PVDF/sodium niobate nanofibrous mat as the active materials for the supercapacitor and nanogenerator device. The individual performance has been examined by fabricating the PVDF/reduced graphene oxide supercapacitor device and PVDF/sodium niobate nanogenerator device, which shows an excellent performance as the energy storage and harvester, respectively. The integrated self-charging power system can charge the supercapacitor device to 0.8 V in 190 s. Collectively, from the obtained results, the novel self-charging power system will paves the way towards the development of polymer-based self-charging system for wearable/portable electronics applications.

## Figures and Tables

**Table 1:** Comparison of the output voltage of various piezoceramic embedded in the polymer matrix.

Series No.	Piezoelectric Material	Output Voltage	Reference
1	(PAA/OA-BTO NP)_n_ thin films	2.5 V	[33]
2	PVDF-TrFE/BiTO	5.0 V	[34]
3	PVDF-TrFE/(Na, K)NbO_3_	2.0 V	[35]
4	PVDF/graphene	12 V	[36]
5	BaTiO_3_+CNT+PDMS	3.0 V	[37]
6	PVDF/Sodium niobate	40 V	This work

**Table 2:** Comparison of the self-powered supercapacitor performances using triboelectric nanogenerator (TENG) and piezoelectric nanogenerator (PENG).

Series No.	Energy Harvester	Energy Storage	Charing Voltage (mV)	Time	Reference
1	Planar type TENG	Planar supercapacitor	800	3 h	[54]
2	Planar type TENG	Planar supercapacitor	250	100 s	[55]
3	Planar type TENG	Kirigami supercapacitor	50	20 s	[56]
4	Planar type TENG	Micro-supercapacitor	400	20 min	[57]
5	Planar PENG	Supercapacitor	2500	12 h	[58]
6	Electrospun PVDF/Sodium niobate PENG	Binder free supercapacitor	800	190 s	This work

## Supplementary Materials

The following are available online at https://www.mdpi.com/2072-666X/11/2/198/s1: Figure S1: Raman spectrum of sodium niobite, Figure S2: FE-SEM and elemental mapping analysis of sodium niobate, Figure S3: Comparative voltage output of bare PVDF and PVDF/sodium niobate subjected to an applied compressive force of 10 N, Figure S4: FE-SEM micrograph of reduced graphene oxide, Figure S5: Coulombic efficiency of the PVDF/reduced graphene oxide SSC as a function of current., Figure S6: Cyclic stability of PVDF/reduced graphene oxide SSC device over 10,000 continuous cycles charge-discharge analysis measured at a current of 50 nA, Figure S7: Schematic representation of the self-charging system with supercapacitor and nanogenerator, Figure S8: The charging profile of the PVDF/reduced graphene oxide SSC with the different applied compressive force applied to the PVDF/sodium niobate nanogenerator device, Figure S9: Comparison of time to charge the PVDF/reduced graphene oxide SSC with the applied compressive force.
