# Supplementary material for: Free-Standing PVDF/Reduced Graphene Oxide Film for All-Solid-State Flexible Supercapacitors towards Self-Powered Systems
Source: Micromachines (Basel). 2020 Feb 14;11(2):198. doi: 10.3390/mi11020198 (PMC7074646; doi:10.3390/mi11020198)
Supplement: Supplementary file 1 [file micromachines-11-00198-s001.pdf]

micromachines

Supplementary Materials: Free-Standing Graphene/PVDF Film for All-Solid-StateF Supercapacitors towards Self-Powered Systems

Parthiban Pazhamalai, Vimal Kumar Mariappan, Surjit Sahoo, and Sang -Jae Kim

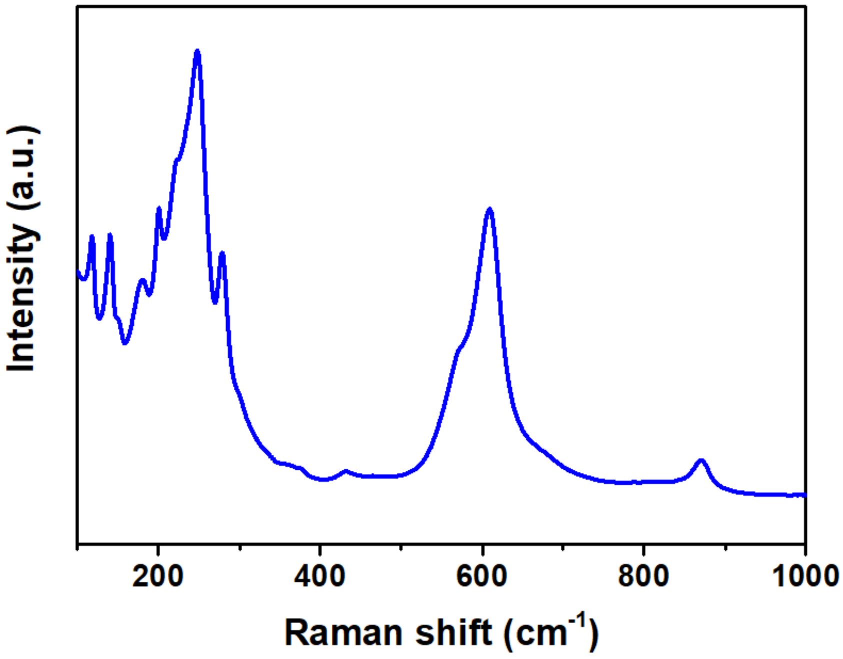

Figure S1. Raman spectrum of sodium niobate.

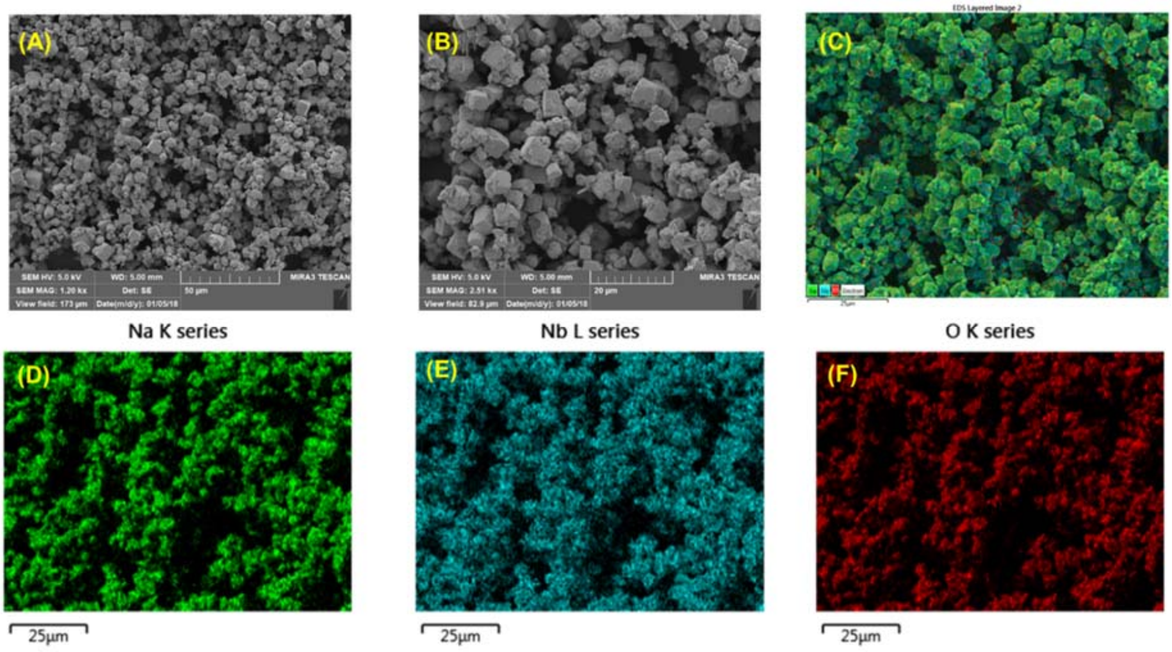

Figure S2. FESEM and elemental mapping analysis of sodium niobate.

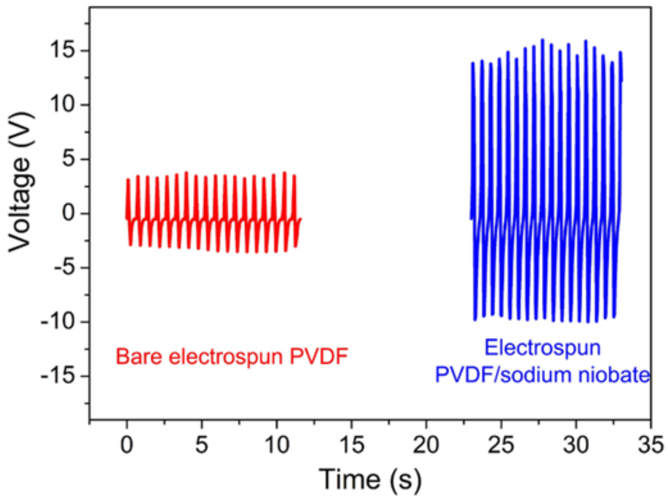

Figure S3. Comparative voltage output of bare PVDF and PVDF/sodium niobate subjected to an applied compressive force of 10 N.

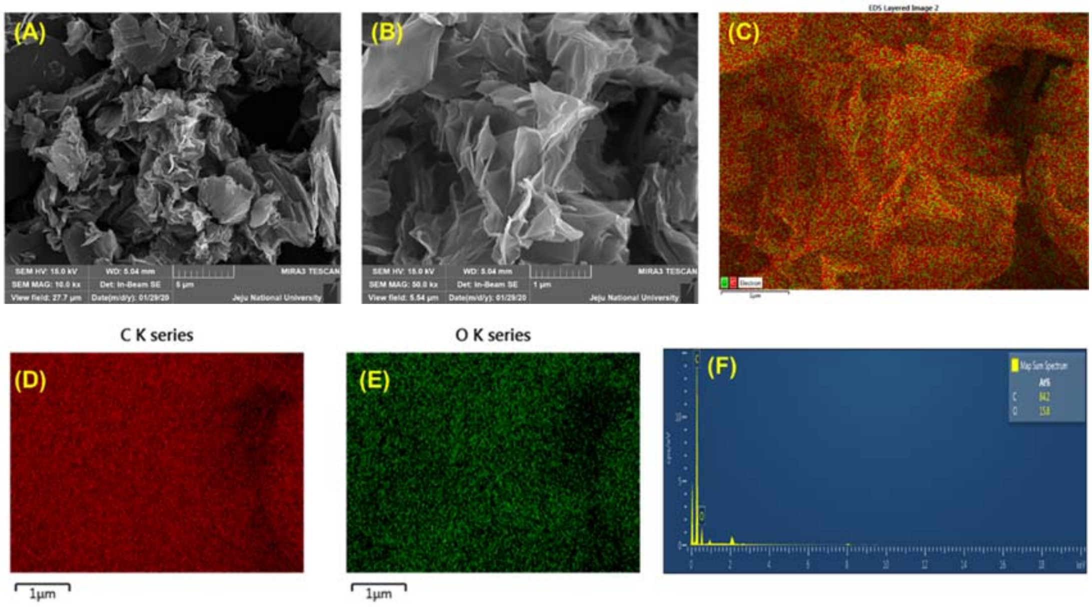

Figure S4. FESEM micrograph of reduced graphene oxide (A) high magnification; (B) low magnification; (C) overlay field emission micrograph, (D) elemental mapping analysis of carbon; (E) elemental mapping analysis of oxygen present in the reduced graphene oxide and (F) EDS spectrum of the reduced graphene oxide.

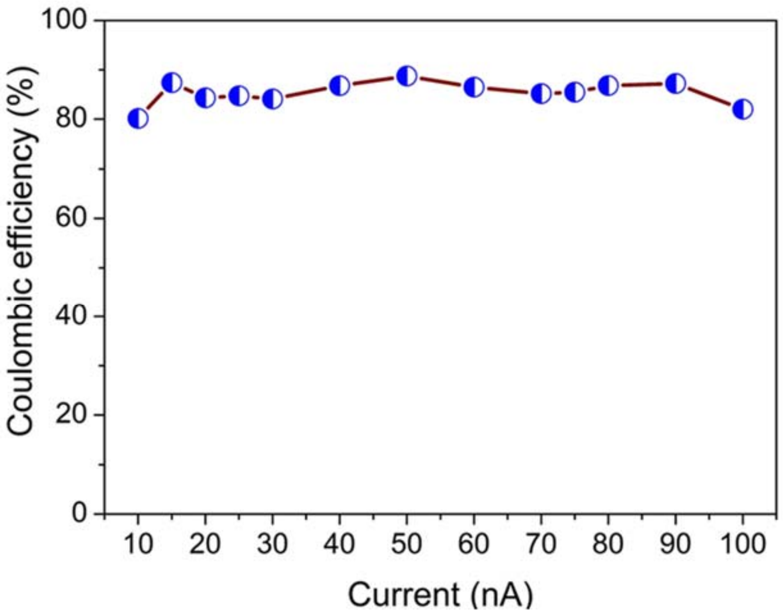

Figure S5. Coulombic efficiency of the PVDF/reduced graphene oxide SSC as a function of current.

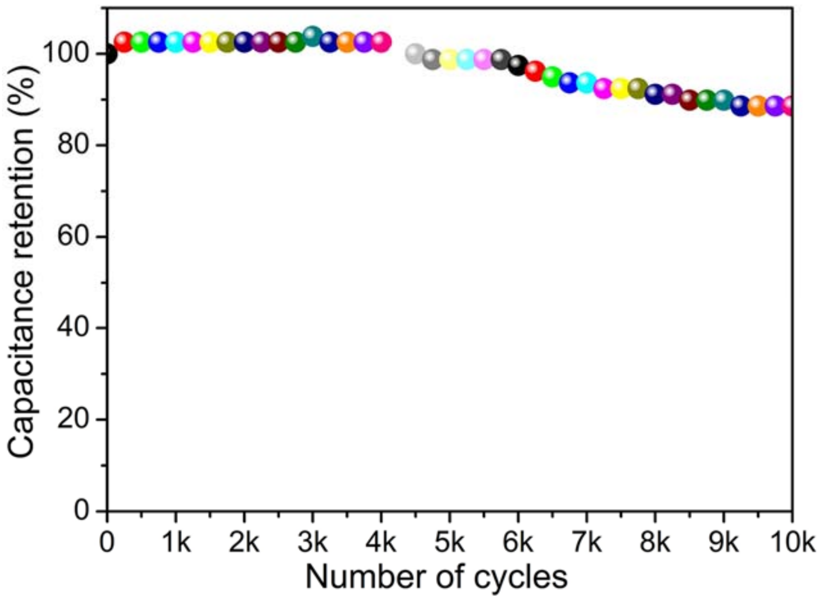

Figure S6. Cyclic stability of PVDF/reduced graphene oxide SSC device over 10,000 continuous cycles charge-discharge analysis measured at a current density of 50 nA.

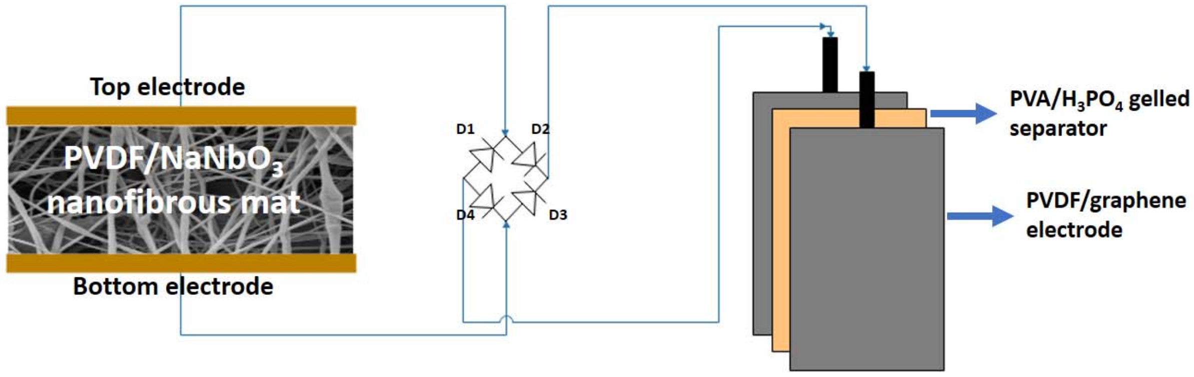

Figure S7. Schematic representation of the self-charging system with supercapacitor and nanogenerator.

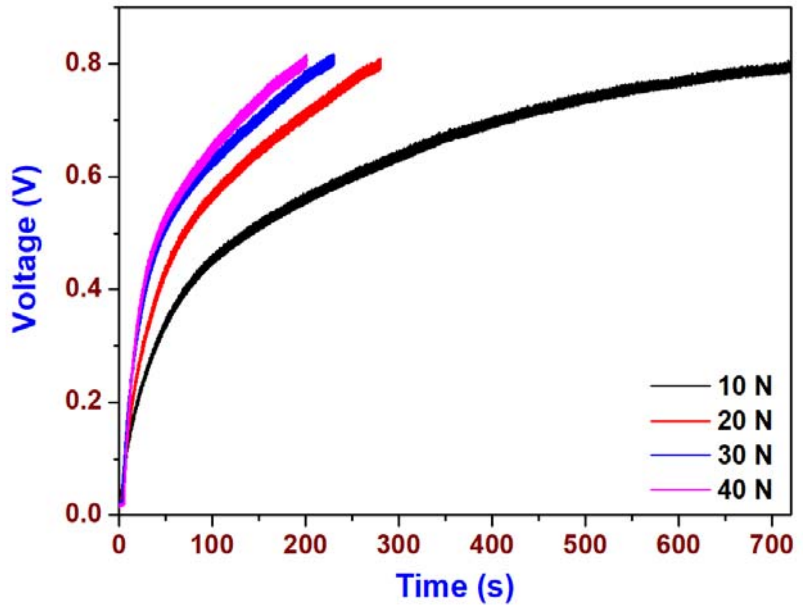

Figure S8. The charging profile of the PVDF/reduced graphene oxide SSC with the different applied compressive force applied to the PVDF/sodium niobate nanogenerator device.

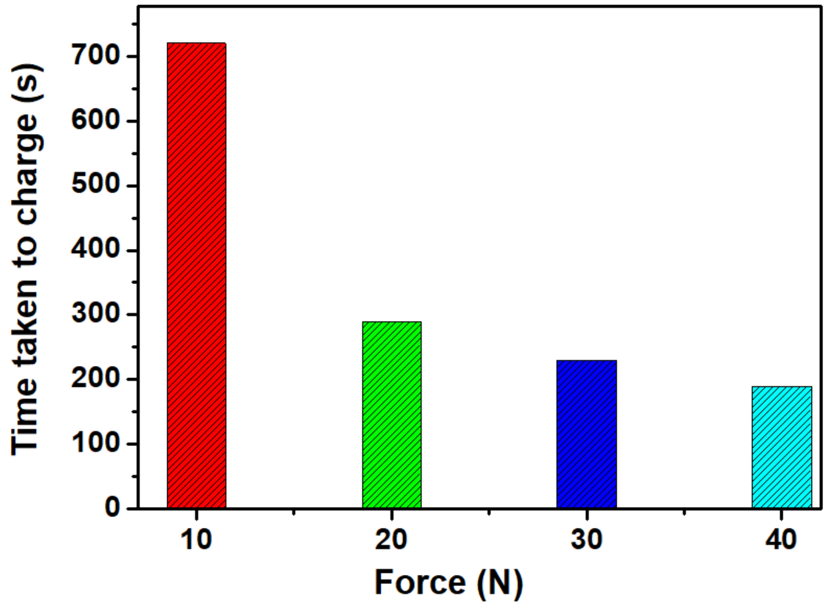

Figure S9. Comparison of time to charge the PVDF/reduced graphene oxide SSC with the applied compressive force.

© 2020 by the authors. Submitted for possible open access publication under the terms and conditions of the Creative Commons Attribution (CC BY) license (http://creativecommons.org/licenses/by/4.0/).
